# The role of mitochondria in eosinophil function: implications for severe asthma pathogenesis

**DOI:** 10.3389/fcell.2024.1360079

**Published:** 2024-03-01

**Authors:** Janice Koranteng, Kian Fan Chung, Charalambos Michaeloudes, Pankaj Bhavsar

**Affiliations:** ^1^ National Heart and Lung Institute, Imperial College London, London, United Kingdom; ^2^ Royal Brompton & Harefield NHS Trust, London, United Kingdom; ^3^ School of Medicine, European University Cyprus, Nicosia, Cyprus

**Keywords:** severe asthma, eosinophils, mitochondria, pathogenesis, metabolism

## Abstract

Mitochondria are key metabolic hubs involved in cellular energy production and biosynthesis. ATP is generated primarily by glucose and fatty acid oxidation through the tricarboxylic acid (TCA) cycle and oxidative phosphorylation (OXPHOS) in the mitochondria. During OXPHOS there is also production of reactive oxygen species (ROS), which are involved in the regulation of cellular function. Mitochondria are also central in the regulating cell survival and death, particularly in the intrinsic apoptosis pathway. Severe asthma is a heterogeneous disease driven by various immune mechanisms. Severe eosinophilic asthma entails a type 2 inflammatory response and peripheral and lung eosinophilia, associated with severe airflow obstruction, frequent exacerbations and poor response to treatment. Mitochondrial dysfunction and altered metabolism have been observed in airway epithelial and smooth muscle cells from patients with asthma. However, the role of mitochondria in the development of eosinophilia and eosinophil-mediated inflammation in severe asthma is unknown. In this review, we discuss the currently limited literature on the role of mitochondria in eosinophil function and how it is regulated by asthma-relevant cytokines, including interleukin (IL)-5 and granulocyte-macrophage colony-stimulating factor (GM-CSF), as well as by corticosteroid drugs. Moreover, we summarise the evidence on the role of mitochondria in the regulation of eosinophils apoptosis and eosinophil extracellular trap formation. Finally, we discuss the possible role of altered mitochondrial function in eosinophil dysfunction in severe asthma and suggest possible research avenues in order to better understand their role in disease pathogenesis, and identify novel therapeutic targets.

## 1 Introduction

### 1.1 Mitochondrial function and dynamics

Mitochondria are membrane-enclosed organelles that contain their own DNA, mitochondrial DNA (mtDNA), and generate most of the cell’s energy in the form of ATP, using glucose, fatty acids and amino acids as energy substrates. Glycolysis, occurring in the cytoplasm, metabolises glucose into pyruvate that is converted into acetyl-coenzyme A (CoA). Glycolytic intermediates are also re-directed into fatty acid and amino acid biosynthesis, and into the pentose phosphate pathway that generates nicotinamide adenine dinucleotide phosphate (NADPH) required for antioxidant protection (Michaeloudes et al., 2020). Acetyl-CoA, also generated by fatty acid oxidation, feeds into the tricarboxylic acid cycle (TCA) in the mitochondrial matrix, which produces the reducing intermediates reduced nicotinamide adenine dinucleotide (NADH) and reduced flavin adenine dinucleotide (FADH_2_) (Michaeloudes et al., 2020). Reducing intermediates transfer electrons to molecular oxygen through redox reactions, catalysed by a series of electron-carrying complexes (complex I-IV), termed the electron transport chain, in a process called oxidative phosphorylation (OXPHOS), which occurs in the inner mitochondrial membrane. OXPHOS creates a membrane potential (ΔΨm) that powers the production of ATP and generates reactive oxygen species (ROS) (Michaeloudes et al., 2017). Cellular ROS can also be generated through the activity of other enzymes, such as NADPH oxidases (NOX) during oxidative respiratory burst in immune cells (Michaeloudes et al., 2021). ROS participate in cell signalling and inter-organelle communication, however, excess amounts of ROS, can cause DNA damage and activate redox-sensitive inflammatory pathways, resulting in cell death and inflammation (Prakash et al., 2017). Cristae are folds within the inner mitochondrial membrane that increase the surface area of the inner membrane, allowing for greater and faster ATP production. Their volume and remodelling can directly relate to the respiratory processes of the cell (Bonjour et al., 2022). Mitochondria also mediate the intrinsic pathway of apoptosis. Pro-apoptotic mediators interact with the mitochondria, causing the opening of the mitochondrial permeability transition pore (MPTP), loss of mitochondrial membrane potential and release of cytochrome *c* and other pro-apoptotic proteins into the cytosol, leading to caspase activation and cell dismantling (Druilhe et al., 2003). This differs from extrinsic apoptosis which works through receptor-mediated pathways, for example, Fas ligand binding to Fas receptor. This triggers the activation of Fas-associated death domain (FADD), death-inducing signaling complex (DISC) and caspase-8. The perforin/granzyme pathway of apoptosis involves the release of cytoplasmic granules (granzyme A and granzyme B) through the transmembrane pore perforin. This activates caspase 10 or leads to the formation of the nucleosome assembly protein SET complex and DNA degradation. All three pathways of apoptosis lead to the execution pathway; the final stage of apoptosis initiated by activation of caspase-3, followed by morphological changes such as nuclear fragmentation and phosphatidylserine externalization as a signal for phagocytosis of apoptotic cells. Necrosis can occur in response to stress stimuli and causes disruption of cellular membrane and release of cytoplasmic contents (Elmore, 2007).

Cells can adapt to stress and dysfunction through changes in their metabolism, and coordinated regulation of mitochondrial number and morphology (Eisner et al., 2018). Mitochondrial biogenesis involves division of pre-existing mitochondria and is regulated by the peroxisome proliferator-activated receptor gamma co-activators (PGC)-1α and β (Ventura-Clapier et al., 2008). Mitochondria can be found segregated or in networks as a result of fission or fusion, respectively, mediated by dynamin-related GTPases (Youle and van der Bliek, 2012). Mitochondrial fusion is mediated by mitofusins 1 and 2 (Mfn1, Mfn2), optic atrophy protein (Opa1), and fission by dynamin-related protein (Drp 1). Mild stress can induce mitochondrial biogenesis and fusion, which allows complementation between mitochondria protecting them from damage. Prolonged or severe stress promotes segregation of damaged mitochondria through fission, allowing them to be removed through autophagy (mitophagy) (Michaeloudes et al., 2017). Activation of glycolysis is important in immune cell activation and survival under conditions of inflammation and stress, as it generates intermediates required for the biosynthesis of the antioxidant glutathione, nucleotides and lipids required for the production of inflammatory mediators (Michaeloudes et al., 2020). mtDNA encode the 12S and 16S ribosomal RNA genes and 22 transfer RNA genes that are needed for the synthesis of mitochondrial proteins and also encode 13 polypeptides that are essential for components of the ETC. Genetic changes and polymorphisms in mtDNA including mitochondrial haplotype U and mitochondrial MELAS A3243G, have been associated with asthma (Reddy, 2011).

### 1.2 Severe asthma

Asthma pathology involves chronic inflammation associated with airway structural changes, termed airway remodelling and increased mucus production. Airway remodelling involves increased airway smooth muscle mass and deposition of extracellular matrix, which lead to airway lumen narrowing and airflow limitation. Another clinical feature of asthma is airway hyper-responsiveness, which can lead to exaggerated airway narrowing in response to environmental irritants (Barnes, 2017).

The asthmatic immune response is heterogeneous and depends on multiple factors, such as atopic status and environmental exposures. A large proportion of patients, predominantly atopic, show type 2 inflammation involving a T helper type 2 (Th2) immune response, eosinophilia and basophil and mast cell activation. This response is driven by chronic activation and injury of the airway epithelium by allergens, leading to loss of integrity and release of damage-associated molecular patterns and cytokines (Raby et al., 2023). The alarmins IL-25, IL-33 and thymic stromal lymphopoietin (TSLP) activate dendritic cells, and induce the release of the Th2 cytokines IL-4, IL-13 and IL-5, and other mediators, by type 2 innate lymphoid cells (ILC2) leading to eosinophil recruitment and activation (Whetstone et al., 2022). Activated dendritic cells drive T cell differentiation into Th2 cells, which promote eosinophil infiltration and activation, and induce B cells to produce IgE. IgE triggers mast cell degranulation and release of histamine and other mediators promoting airway smooth muscle contraction (Barnes, 2017). The damaged epithelium and infiltrating immune cells produce growth factors and inflammatory mediators that drive airway remodelling, and goblet cell hyperplasia and increased mucus production (Raby et al., 2023). On the other hand, neutrophilic asthma, associated with lung infections, air pollution and obesity, is driven by a Th1/Th17 immune response (De Volder et al., 2020).

For most asthma patients, symptoms can be effectively controlled by inhaled treatment with corticosteroids in combination with long-acting beta agonists, whilst long-acting muscarinic antagonists and leukotriene inhibitors maybe added for the treatment of poorly-controlled patients (Chung et al., 2022). However, a small proportion of patients, suffering from severe asthma, cannot control their symptoms using these medications. These patients have frequent exacerbations, are on a high-dose of inhaled corticosteroids and frequently use oral corticosteroids despite ongoing symptoms (Chung, 2013). Prolonged use of high doses of inhaled and oral corticosteroids leads to significant side effects, including increased risk of infections, dyslipidaemia, hypertension and osteoporosis (Ora et al., 2020). New more effective therapies for severe asthma are therefore crucial. Severe asthma is a heterogeneous disease, entailing diverse clinical phenotypes associated with specific inflammatory and other molecular phenotypes. Current research efforts aim at using a systems biology approach to identify molecular mechanisms driving the different clinical phenotypes in order to define disease endotypes and design therapies targeted to individual patients. The Unbiased Biomarkers in Prediction of Respiratory Disease Outcomes (U-BIOPRED) consortium identified three molecular phenotypes in severe asthma: T2-high eosinophilic, and T2-low neutrophilic and paucigranulocytic asthma. The same study demonstrated that sputum cells from patients with paucigranulocytic asthma show enrichment for OXPHOS genes (Kuo et al., 2017). These findings highlight the importance of understanding the metabolic phenotype of immune cells for defining severe asthma endotypes.

### 1.3 Severe eosinophilic asthma

Patients with severe eosinophilic asthma show a type 2 inflammatory response, severe airflow obstruction and frequent exacerbations, and are dependent on oral corticosteroids. Severe eosinophilic asthma is defined by an increased number of eosinophils in the blood (≥300 cells/μL) and/or in the airways (sputum eosinophil count ≥3%) (Chung et al., 2022).

Eosinophils are the immune system’s defence against parasitic and helminth infections however, they also act as antigen presenting cells and play a role in antiviral immunity in the lungs (Akuthota et al., 2010; Samarasinghe et al., 2017). Nonetheless, lung eosinophilia promotes pathogenesis of severe asthma and allergic diseases. Under physiological conditions, eosinophils differentiate from pluripotent progenitors in the bone marrow and are released in small numbers into the peripheral blood for a short period (8–18 h) before they migrate into the tissues, primarily in the gastrointestinal tract (Rosenberg et al., 2013a). The lifespan of eosinophils in tissues is thought to be 2–5 days. This is similar to the lifespan of neutrophils, which is estimated to be 8–20 h in the circulation and 1–4 days in tissues (Luo and Loison, 2008). Eosinophilopoiesis is dramatically increased as a result of Th2 cell responses associated with allergic diseases including asthma (Rothenberg and Hogan, 2006; Rosenberg et al., 2013a; Travers and Rothenberg, 2015). This increase in eosinophil production is driven by a dedicated set of cytokines, namely, IL-3, IL-5, and granulocyte-macrophage colony-stimulating factor (GM-CSF) (Rothenberg and Hogan, 2006; Rosenberg et al., 2013a). IL-5 and IL-3 are produced by Th2 cells, as well as mast cells and eosinophils themselves. GM-CSF is primarily produced by T cells, macrophages and epithelial cells (Broughton et al., 2012). Amongst these, the Th2-associated cytokine IL-5 is the most specific cytokine for the eosinophil lineage and is responsible for the expansion of eosinophils from their bone marrow progenitors, their release into the blood and their survival following migration into the tissues (Lopez et al., 1988; Rothenberg and Hogan, 2006; Rosenberg et al., 2013a). Eotaxins (CCL11 and CCL24) are considered to be important chemokines involved in the recruitment of eosinophils into the airways while IL-4 and IL-3 are reported to be overexpressed in the airways of severe asthmatics (Wenzel et al., 2007; Wenzel et al., 2013). Recruitment of eosinophils from the circulation requires blood eosinophils to become activated, leading to their attachment to activated endothelium and their extravasation into the airway wall through the bronchial tissue and epithelium into the airway lumen (Barthel et al., 2008; Johansson and Mosher, 2013). Blood eosinophils from subjects with allergy or asthma have a greater degree of adhesion and trans-endothelial migration, and greater responsiveness to chemo-attractants compared to those from normal volunteers (Håkansson et al., 1997). At the site of injury, eosinophils can release their cytotoxic granule proteins, a process called degranulation, as well as cytokines, chemokines and lipid mediators, such as CCL11, CCL5, CCL3, leukotrienes, and platelet activating factor (PAF). This causes exacerbation of inflammation and tissue damage, which is particularly deleterious when Th2 responses are directed against allergens (Rothenberg and Hogan, 2006; HOGAN et al., 2008; Rosenberg et al., 2013b). Inhaled and oral corticosteroids reduce blood and sputum eosinophil numbers in patients with asthma (Lazarus et al., 2007; Dente et al., 2010; Lommatzsch et al., 2019; Prazma et al., 2019).

Anti-IL-5 biologic therapy has been developed to treat these patients, as IL-5 is the primary cytokine involved in eosinophil survival. Mepolizumab binds to and neutralises IL-5, leading to a reduction in both blood and airway eosinophil numbers due to the inhibition of IL-5 mediated cell survival (Chung et al., 2022). Benralizumab targets the IL-5 receptor alpha (IL-5Rα) on eosinophils, causing a depletion in eosinophil numbers via antibody-dependent cell-mediated cytotoxicity (ADCC) (Chung, 2013). Severe eosinophilic asthmatics on anti-IL-5 biologic therapy have shown an improvement in asthma symptoms, with a reduction in the number of exacerbations (Chung et al., 2022; Chung, 2013). Nonetheless, some patients do not adequately respond to biologic therapies, therefore, it is important to identify new therapeutic targets for reducing eosinophilia in severe asthma patients.

### 1.4 Mitochondrial dysfunction and asthma

Altered mitochondrial function and cellular metabolism have been identified in asthma, where they may contribute to disease pathology. Studies using animal models of allergen-induced lung inflammation have shown that mitochondrial dysfunction, involving altered mitochondrial ROS production, morphology and mitophagy, may drive asthma pathology (Zhang et al., 2020; Bruno et al., 2021). However, there is limited evidence on mitochondrial function in immune or structural cells from patients with asthma. Peripheral blood mononuclear cells from severe asthmatics show increased OXPHOS and cellular ROS in comparison to healthy volunteers (Ederlé et al., 2019). Airway smooth muscle cells from patients with asthma show augmented mitochondrial fission associated with increased Drp1 and decreased Mfn2 expression (Aravamudan et al., 2014). Furthermore, airway smooth muscle cell hyperplasia in severe asthma is driven by augmented OXPHOS, resulting from mitochondrial biogenesis and a switch towards using fatty acids as a metabolic substrate (Trian et al., 2007a; Esteves et al., 2021). Increased OXPHOS, driven by increased arginine metabolism, has also been identified in airway epithelial cells, where it has a protective effect (Xu et al., 2016a). The increased mitochondrial metabolism observed in asthmatic airways may be a result of an adaptive response to cellular damage caused by oxidative stress and inflammation. Indeed, studies in allergic asthma mice models show oxidative damage of mitochondria in the bronchial epithelium (Mabalirajan et al., 2008).

Mitochondria play a central role in the regulation of eosinophil apoptosis, as IL-5 and GM-CSF promote eosinophil survival by inhibiting the mitochondrial (intrinsic) pathway of apoptosis (Kikly et al., 2000). IL-5 and GM-CSF also increase mitochondrial and glycolytic respiration of eosinophils in atopic and non-atopic subjects (Bao et al., 2014). However, the role of glycolysis and mitochondrial metabolism in eosinophil survival and activation in severe asthma is currently unclear.

In this review, we aim to summarise the limited literature currently available on eosinophil mitochondria, particularly their structure and dynamics, role in apoptosis and cellular metabolism and how this facilitates their cellular function. We will then discuss the possible implications this may have in severe eosinophilic asthma, as the role of eosinophil mitochondrial function has not yet been explored in this disease.

## 2 Regulation of mitochondrial structure and function in eosinophils

Human eosinophils contain a small number of mitochondria, approximately 24–36 per cells, compared to other cell types such as lung fibroblasts and macrophages, reported to contain approx. 300 and 700 mitochondria per cell, respectively (Robin and Wong, 1988; Peachman et al., 2001). On the other hand, eosinophils have more mitochondria and a higher mitochondrial respiration rate compared to neutrophils, reported to have only five to six mitochondria per cell (Peachman et al., 2001). They also show a more sustained upregulation of OXPHOS in response to phorbol myristate stimulation, suggesting a greater ability to adapt their metabolic activity to extracellular stimuli. The glycolytic activity of eosinophils, on the other hand, is similar to that of neutrophils, despite neutrophils having more glycogen stores (Porter et al., 2018).

Mitochondrial dynamics and respiration in eosinophils are regulated during their differentiation and in response to their microenvironment. A study in bone marrow-derived eosinophils demonstrated that during eosinophil maturation there is a marked reduction in mitochondrial number and size, which could be due to removal of mitochondria by mitophagy. Furthermore, mature eosinophils exhibited fewer inter-mitochondrial, mitochondria-secretory granules and mitochondrial-ER contacts (Bonjour et al., 2022).

Studies in allergen-induced mouse models of asthma have identified different subtypes of eosinophils, namely, resident eosinophils and recruited inflammatory eosinophils (Mesnil et al., 2016; Wiese et al., 2023). A recent study characterised the metabolic profile of these eosinophil subsets and found that inflammatory eosinophils relied more on glycolytic respiration, whereas resident eosinophils were more dependent on OXPHOS and had higher number of mitochondria (Andreev et al., 2021). Increased glycolysis in inflammatory eosinophils may support the demands of activated eosinophils for the biosynthesis of inflammatory lipid and protein mediators, and for maintaining redox balance. On the other hand, predominance of OXPHOS for energy production may provide the energy required for the sustained survival of resident eosinophils.

**FIGURE 1:**
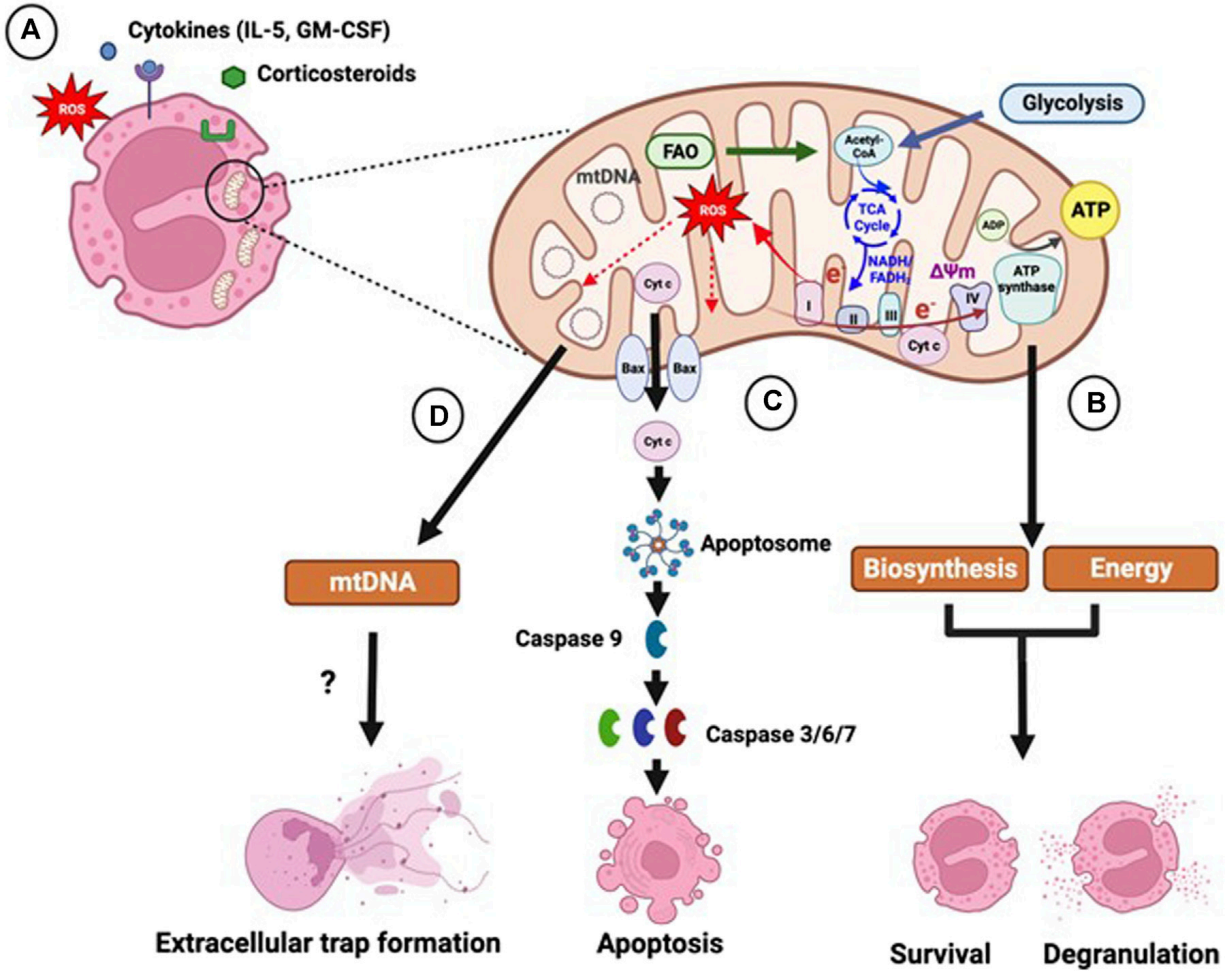
Role of mitochondria in eosinophil function. **(A)** Eosinophils contain a small number of mitochondria, whose activity is regulated by extracellular factors, such as pro-inflammatory cytokines, reactive oxygen species (ROS) and corticosteroid drugs. **(B)** Mitochondria are key sites of energy production and biosynthesis. Acetyl-coenzyme A (Acetyl-CoA) generated by glycolysis and fatty acid oxidation (FAO) enters the tricarboxylic acid (TCA) cycle, which generates the reducing intermediates reduced nicotinamide adenine dinucleotide (NADH) and reduced flavin adenine dinucleotide (FADH_2_). Electrons (e^−^) from the reducing intermediates are transferred to molecular oxygen through a series of electron-carrying protein complexes (complex I-IV), termed the electron transport chain. The movement of electrons generates energy, which facilitates the movement of protons into the intermembrane space leading to the development of a membrane potential (ΔΨm) across the inner membrane. ΔΨm drives the re-entry of protons back into the mitochondrial matrix through the ATP synthase complex driving the phosphorylation of ADP to ATP. Electron leakage during OXPHOS can lead to generation of mitochondrial ROS. Glycolytic and TCA cycle intermediates, and acetyl-CoA, are used as precursors for fatty acid and amino acid synthesis. Increased glycolytic, TCA cycle and OXPHOS activity in response to inflammatory cytokine stimulation may therefore support eosinophil survival and activation by providing biosynthetic intermediates and energy. **(C)** Mitochondria are central in the intrinsic apoptosis pathway, which is induced by cellular stress. Intrinsic apoptosis entails the mitochondrial translocation and oligomerization of Bcl-2 pro-apoptotic proteins, such as Bax, which form pores in the mitochondrial membrane leading to the release of pro-apoptotic factors, including cytochrome c, inducing the formation of the protein complex apoptosome. The apoptosome triggers the activation of the initiator caspase 9 and subsequently of the effector caspases 3, 6 and 7 promoting apoptosis. Corticosteroids trigger mitochondrial-dependent eosinophil apoptosis, which is inhibited by cytokines, such as interleukin (IL)-5 and granulocyte macrophage colony-stimulating factor (GM-CSF). **(D)** Mitochondria may contribute to the formation of eosinophil extracellular traps, which facilitate the capture and killing of bacteria. Extracellular traps are composed of DNA and granule proteins and are released by either live eosinophils or during cell death. Extracellular traps produced by live eosinophils have been shown to contain mitochondrial DNA (mtDNA); however other studies show that the DNA is solely of nuclear origin. Figure created using Biorender.com.

A study by Jones et al demonstrated that IL-5 and GM-CSF induce glycolysis, OXPHOS and TCA cycle activity in blood eosinophils from both atopic and non-atopic subjects. IL-5-induced OXPHOS and glycolysis in eosinophils is signal transducer and activator of transcription 5 (STAT5) and phosphoinositide 3-kinase (PI3K)/Akt-dependent, with STAT5 and PI3K driving both OXPHOS and glycolysis, whilst Akt contributing only to glycolytic activation. NOX-derived ROS also promotes increased OXPHOS, and TCA cycle activity that provides intermediates for amino acid synthesis. These findings suggest a mechanism of coupling eosinophil activation and degranulation with ATP production and macromolecule biosynthesis (Jones et al., 2020). Apart from its bioenergetic role, ATP has been also shown to promote neutrophil degranulation and phagocytosis through activation of purinergic signalling, a mechanism that may also be important in eosinophils Figure 1 (Bao et al., 2014). The effect of lung inflammation on eosinophil mitochondrial dynamics has also been shown using an allergen-induced mouse model of asthma. Bone marrow-derived eosinophils from these mice showed an increase in the number and volume of mitochondrial cristae, as well as an altered cristae structure Mitochondrial cristae show remodelling into a mix of lamellar structures (parallel linear shapes) and tubular structures (small circular shapes), whereas lamellar only structures of cristae were found in eosinophils from controls. Increased mixed cristae were also seen in mature eosinophils in comparison to immature eosinophils (Bonjour et al., 2022). These changes in mitochondria structure may facilitate the increased OXPHOS activity observed in eosinophils in response to inflammatory stimulation.

Lymphoid tissue eosinophils from an allergic asthma mouse model showed a reduction in mitochondrial respiration and glycolysis in response to influenza A virus, compared to uninfected cells (LeMessurier et al., 2020). This report is in line with findings by Bonjour et al. (2022) showing that eosinophils infected with H1N1 influenza virus had reduced mitochondrial area, cristae number and volume and mitochondria-to-mitochondria interactions compared to uninfected cells. In contrast, lung epithelial cells from chickens infected with H6N2 influenza virus show upregulation of OXPHOS-related genes and increased mitochondrial respiration. Furthermore, increased mitochondrial respiration in chicken lung epithelial cells was shown to facilitate influenza virus replication (Meyer et al., 2017). The attenuated mitochondrial respiration in eosinophils following viral infection may, therefore, be a mechanism for preventing viral replication and allowing them to exert their anti-viral function.

The OXPHOS and glycolytic activity of blood eosinophils was shown not to be different in atopic, compared to non-atopic, subjects (Jones et al., 2020). However, it is currently unknown whether eosinophils from patients with asthma, and particularly severe asthma, show altered mitochondrial function.

## 3 Role of mitochondria in eosinophil apoptosis

Under normal conditions circulating eosinophils have a short life-span undergoing spontaneous apoptosis within a few days, which can be accelerated or inhibited by inflammatory mediators and pharmacological agents (Walsh, 2013). Homeostatic eosinophil apoptosis occurs via the extrinsic pathway that involves activation of receptors, such as the Fas/CD95 death receptor or via the mitochondrion-dependent (intrinsic) pathway. However, intrinsic apoptosis can be induced by cellular stress, such as oxidative stress, calcium overload or DNA damage and is facilitated by the Bcl-2 proteins. This involves inactivation of anti-apoptotic Bcl-2 proteins, including Bcl-2, Bcl-xL and Mcl-1L and the translocation and oligomerization of pro-apoptotic proteins, such as Bax and Bid, within the mitochondrial membrane. These proteins form pores in the mitochondrial membrane leading to mitochondrial membrane permeabilization, loss of ΔΨm and release of cytochrome *c* and other pro-apoptotic proteins into the cytosol (Ilmarinen et al., 2014). Opening of the MPTP in response to calcium overload or ROS also triggers mitochondrial membrane permeabilization (Gardai et al., 2003; Ilmarinen-Salo et al., 2012). Cytochrome c drives the formation of the apoptosome, a protein complex that activates the initiator caspase 9, which in turn induces effector caspases 3, 6 and 7 leading to apoptosis Figure 1 (Ilmarinen et al., 2014). Activation and mitochondrial translocation of Bid has also been observed in Fas ligand-induced eosinophil apoptosis, enhancing the suggestion that mitochondria also play a role in extrinsic apoptosis (Segal et al., 2007). Apoptosis ensures that the cell membrane remains intact, preventing the release of immunogenic and cytotoxic mediators that may induce inflammation. Apoptotic bodies are subsequently cleared by phagocytosis (Alessandri et al., 2011; Walsh, 2013). Natural Killer (NK) cells can also induce apoptosis and clearance of eosinophils through cell-mediated cytotoxicity. This process is partially inhibited in the presence of the electron transport chain inhibitors rotenone and antimycin, suggesting that mitochondrial respiration and mtROS play a role in this process (Awad et al., 2014).

There is evidence of impaired apoptosis in blood eosinophils from patients with asthma, which may contribute to lung eosinophilia. This may, at least partly, be due to the effect of pro-survival cytokines, including IL-3, IL-5 and GM-CSF, which inhibit apoptosis and prolong eosinophil survival (Kankaanranta et al., 2000). In contrast, neutrophil survival is only induced by GM-CSF, but not by IL-3 and IL-5 (Didichenko et al., 2008).

## 4 Inhibition of mitochondrial-induced eosinophil apoptosis by pro-survival cytokines

The IL-5, GM-CSF and IL-3 receptors belong to the type I cytokine receptor family and entail a cytokine-specific α-chain, and a β-chain that mediates signal transduction and is common for all three cytokines (Geijsen et al., 2001). Upon ligand binding, the receptor β-chain dimerizes and leads to activation of the protein tyrosine kinases Janus activated kinase 2 (Jak2) and Lyn, which in turn induce the transcription factors signal transducer and activator of transcription (STATs), and the Ras–mitogen-activated protein kinase (MAPK) pathway. These pathways mediate the pro-survival effects of these cytokines on eosinophils (Park and Bochner, 2010).

IL-5-mediated activation of the Jak-STAT cascade leads to inhibition of the intrinsic pathway of apoptosis in eosinophils by preventing the translocation of the Bax protein to the mitochondria. This effect may be mediated by upregulation of the anti-apoptotic protein Bcl-xL (Dibbert et al., 1998; Dewson et al., 2001). Furthermore, IL-5 and GM-CSF-dependent activation of the MAKP Erk1/2 leads to phosphorylation of Bax on threonine residues, facilitating its binding to the peptidyl-prolyl isomerase Pin1. Pin1 inhibits apoptosis by constraining Bax in an inactive form and prevents the exposure of its N-terminal activation domain, leading to inhibition of its activation and mitochondrial translocation (Shen et al., 2009).

Regulation of cell death may also depend upon the activation state of eosinophils. Sialic acid-binding immunoglobulin-like lectin (Siglec)-8 is a glycan cell surface receptor expressed on human mast cells, basophils and eosinophils (Kikly et al., 2000). Cross-linking Siglec-8 with monoclonal antibodies has been shown to induce eosinophil apoptosis, and to decrease eosinophilic inflammation and airway remodelling in allergen-exposed mice (Nutku et al., 2003; Song et al., 2009). Intriguingly, IL-5- or GM-CSF-primed eosinophils have been shown to be more sensitive to Siglec-8-induced apoptosis (Nutku et al., 2003). A subsequent study reported that cross-linking with an anti-Siglec-8 antibody induced predominantly apoptosis in IL-5-naïve, and necrosis in IL-5-primed, eosinophils. This may be caused by Siglec-8 antibody-induced ROS augmenting and prolonging the activation of Erk1/2 by IL-5 (Kano et al., 2013). These findings suggest that activated eosinophils may be undergoing necrosis, a more immunogenic cell death, compared to resting eosinophils that undergo apoptosis.

## 5 Corticosteroid-induced eosinophil apoptosis

Corticosteroids, such as dexamethasone, induce the mitochondrial pathway of apoptosis in eosinophils, which contributes to the resolution of airway inflammation (Ora et al., 2020). The effects of corticosteroids on cell function are exerted via the glucocorticoid receptor (GR). Following corticosteroid binding, GR dimerizes and translocates to the nucleus where it binds to glucocorticoid-responsive elements on promoters of genes, such as the anti-inflammatory mitogen-activated protein kinase phosphatase-1 (MKP-1), leading to their transactivation. GR can also bind to negative GREs on gene promoters inhibiting gene expression, a process termed direct trans-repression. Alternatively, interaction of GR with pro-inflammatory transcription factors, such as NF-κΒ and activating protein (AP)-1, leads to indirect trans-repression, which mediates most of the anti-inflammatory effects of corticosteroids (Milara et al., 2023). Dexamethasone was shown to promote apoptosis of eosinophils from atopic subjects in a concentration-dependent manner, by triggering an early ROS-dependent activation of the MAPK c-Jun NH_2_-terminal kinase (Jnk) leading to increased mitochondrial translocation of Bax. Bid cleavage and subsequent mitochondrial translocation was also shown to mediate dexamethasone-induced apoptosis in peripheral eosinophils from healthy subjects and in eosinophils in bronchoalveolar lavage from allergen-exposed mice (Segal et al., 2007; Maret et al., 2009). Dexamethasone also reduces the expression of the anti-apoptotic X-linked inhibitor of apoptosis protein (XIAP) the mitochondrial antioxidant manganese superoxide dismutase (MnSOD), augmenting mitochondrial ROS generation and loss of ΔΨm, and leads to prolonged Jnk activation (Gardai et al., 2003). Enhanced degradation of the anti-apoptotic protein Mcl-1L has also been shown to contribute to dexamethasone-induced eosinophil apoptosis. In contrast, corticosteroids delay neutrophil apoptosis by stabilising the expression of Mcl-1L protein (Sivertson et al., 2007). GM-CSF was shown to inhibit dexamethasone-induced apoptosis of eosinophils from atopic subjects, by attenuating Bax activation and mitochondrial translocation, and by preventing XIAP inhibition and prolonged Jnk activation (Gardai et al., 2003). Dexamethasone and IL-5 synergistically upregulate the expression of the transcription factor Nuclear Factor IL-3 (NFIL3), leading to attenuated apoptosis of peripheral eosinophils from healthy subjects (Pazdrak et al., 2016). It is suggested that severe asthmatics that are resistant to corticosteroid-induced eosinophil apoptosis, most likely have an increased production of IL-5 and GM-CSF in the circulation, which promotes the survival of eosinophils and inhibits the suppressive action of corticosteroids (Gardai et al., 2003).

## 6 Eosinophil extracellular traps

Eosinophils release eosinophil extracellular traps (EETs) which are composed of DNA and granule proteins such as eosinophil cationic protein (ECP) and major basic protein (MBP). EETs can be released by either live eosinophils or eosinophils undergoing cell death, possibly depending on the stimuli available in the extracellular environment (Yousefi et al., 2008; Morshed et al., 2012; Ueki et al., 2013). Cell death-associated EET formation involves chromatin disassembly and cell membrane lysis, leading to the release of DNA, histones and granule proteins (Ueki et al., 2013). In EETs released by live cells, DNA has been shown to be of mitochondrial origin based on the absence of histone proteins and the expression of mitochondrial DNA genes Figure 1 (Yousefi et al., 2008; Silveira et al., 2019). However, this is disputed due to the small number of mitochondria in eosinophils and the high energy required for release of mtDNA (Mukherjee et al., 2018).

EETs can form networks to capture and kill bacteria, as part of their antibacterial mechanism, but can also cause tissue damage and amplify inflammation under pathogenic conditions (Yousefi et al., 2008; Yousefi et al., 2012). Airway biopsies from patients with mild asthma have shown evidence of increased EET formation, which was not further enhanced by exposure of subjects to allergen (Dworski et al., 2011). A greater proportion of EET-positive eosinophils was observed in the peripheral blood of severe asthma patients, compared to non-severe asthma patients and healthy subjects (Choi et al., 2020). Moreover, peripheral eosinophils from severe asthma patients showed greater EET formation in response to IL-5 or lipopolysaccharide (LPS) stimulation (Choi et al., 2018). Intranasal exposure of mice with EETs showed increased airway inflammation, epithelial thickening and increased the expression of the epithelial-derived cytokines IL-33 and thymic stromal lymphopoietin (TSLP), which activate type 2 innate lymphoid (ILC2) cells to produce Th2 cytokines (Choi et al., 2020). EETs were also shown to act in an autocrine fashion to induce eosinophil degranulation, and to promote loss of airway epithelial barrier function (Choi et al., 2018).

Release of EETs from live cells requires cell adhesion and priming by inflammatory mediators, including IL-5, IFN-γ, eotaxin and thymic stromal lymphopoietin (TSLP) (Yousefi et al., 2008; Morshed et al., 2012). EET formation occurs at a slower rate than degranulation, with maximal levels of mtDNA being reached at 30–60 min, whereas release of eosinophil peroxidase occurs within 1 minute in IL-5 and GM-CSF-primed mouse and human eosinophils stimulated with complement factor 5a (C5a) (Germic et al., 2021). NADPH oxidase (Nox)-mediated ROS is required for the formation of EETs containing mtDNA, as the Nox inhibitor diphenyleneiodonium (DPI) has been shown to inhibit their release from C5a- or TSLP-stimulated blood eosinophils of healthy subjects (Yousefi et al., 2008; Morshed et al., 2012). In line with these findings, ROS-deficient eosinophils from chronic granulomatous patients were unable to form EETs, after priming with IL-5 or IFN-γ and stimulating with LPS (Yousefi et al., 2008). ROS-dependent EETs formation was also observed in airway eosinophils from a mouse model of allergen-induced airway inflammation (Silveira et al., 2019).

The role of mitochondria in EET formation is incompletely understood. mtDNA has pro-inflammatory effects by activating Toll-like receptor 9 (TLR 9), the NOD-like receptor family, pyrin domain containing 3 (NLRP3) inflammasome, and the stimulator of interferon genes (STING) pathway (Wu et al., 2019; Liu et al., 2021; Dimasuay et al., 2023). In neutrophils from patients with systemic lupus erythromatosus mitochondrial ROS oxidise mtDNA, leading to the formation of extracellular traps containing oxidised mtDNA. Oxidised mtDNA promotes a stronger inflammatory response, than non-oxidised mtDNA, amplifying the immune response (Lood et al., 2016). The relative contribution of Nox- and mitochondrial-derived ROS in extracellular trap formation is unclear; however, mitochondria may be a source of ROS in situations where Nox activity is reduced. Understanding the mechanisms underlying EET formation and the role of mitochondria in this could be important for identification of new therapeutic targets.

## 7 Research outlook

Altered mitochondrial function has been reported in asthma. Specifically, airway smooth muscle cells and epithelial cells from patients with asthma show increased mitochondrial biogenesis and respiration, which may drive abnormal cellular phenotype (Trian et al., 2007b; Xu et al., 2016b; Esteves et al., 2021). Mitochondria play a key role in eosinophil function, particularly in their survival and EET formation Figure 1 (Yousefi et al., 2012; Ilmarinen et al., 2014). Therefore, dysregulated mitochondrial function may also contribute to eosinophilia and airway dysfunction in severe asthma. However, there are currently no published studies on mitochondrial function in eosinophils from patients with severe asthma.

Our ongoing studies show that eosinophils isolated from severe eosinophilic asthma patients show an inherently different metabolic phenotype, involving increased mitochondrial respiration and glycolytic activity (Koranteng et al., 2023)**.** Increased metabolic respiration may be a result of changes in mitochondrial number or morphology, usage of metabolic substrates or altered metabolic pathway activity. Changes in mitochondrial and metabolic gene expression, possibly due to epigenetic changes, may underlie the metabolic reprogramming observed in severe asthma eosinophils. IL-5 and GM-CSF can also promote a similar phenotype of increased mitochondrial respiration and glycolysis in healthy eosinophils (Jones et al., 2020). Furthermore, oxidative stress promotes metabolic re-programming of immune cells, whilst corticosteroids are also known to regulate mitochondrial gene expression and function (Garcia et al., 2003; Previte et al., 2017). Therefore, it is possible that prolonged exposure to high levels of inflammatory mediators, ROS and/or to drugs (particularly corticosteroids) *in vivo* may shape the metabolic activity of severe asthma eosinophils.

Increased survival, as well as a greater capacity for adhesion and trans-endothelial migration of blood eosinophils, contribute to airway eosinophilia in severe asthma. Eosinophils promote airway pathology by inducing epithelial injury and amplifying inflammation through the release of cytokines and chemokines (Rothenberg and Hogan, 2006; HOGAN et al., 2008; Rosenberg et al., 2013b). Mitochondrial function plays a key role in eosinophil survival. Mitochondria are the main source of ATP required for energy-demanding processes such as migration and mediator secretion, whilst ROS are also important in inflammatory mediator production through activation of redox-sensitive signalling pathways (Michaeloudes et al., 2021). Glycolysis generates intermediates for antioxidant protection and macromolecule synthesis required for survival, and lipids needed for production of inflammatory mediators (Michaeloudes et al., 2020). Gene expression can also be affected by metabolic reprogramming through changes in metabolites that act as substrates for epigenetic modifications (Michaeloudes et al., 2020). Transmigration, in severe asthma, exposes the eosinophil to a more hypoxic and nutrient-poor environment. Therefore, eosinophils may undergo metabolic reprogramming in order to adapt and survive in lung parenchyma. To this end studies need to determine whether lung eosinophils show a different metabolic phenotype compared to blood eosinophils. This may lead to a better understanding of the adaptation mechanisms eosinophils use to survive in lung tissue and highlight potential targets for reducing the number of lung eosinophils.

Molecular phenotyping of severe asthma patients has been invaluable in developing novel targeted therapies and is the key to personalised medicine. A greater understanding of the role and mechanisms by which eosinophils can drive this very common endotype of asthma, focusing on aspects of metabolic function will provide a step-change in our understanding of how an altered metabolic phenotype in eosinophils contributes to their persistence in disease by altering the temporal activation of apoptosis and will determine the preference for the source of fuel (glucose, fatty acids or nucleotides) used to generate ATP under basal and stressor conditions. This research will be facilitated by the rapid development of metabolomics technology, and the availability of a wide range of tools for studying different aspects of mitochondrial function, including biogenesis, morphology, ΔΨm, and ROS production. A crucial tool for mitochondrial studies in eosinophils is the Seahorse XF Analyzer, which allows the real-time analysis of OXPHOS activity, glycolysis and metabolic substrate preference.

Identification of the metabolic pathways driving abnormal eosinophil function, can be exploited to develop novel drug therapies targeting glycolytic and mitochondrial dysfunction and airway inflammation in severe asthma.

## References

[B1] Akuthota P. Wang H. Weller P. F. (2010). Eosinophils as antigen-presenting cells in allergic upper airway disease. Curr. Opin. Allergy Clin. Immunol. 10 (1), 14–19. 10.1097/ACI.0b013e328334f693 19949323 PMC2865844

[B2] Alessandri A. L. Duffin R. Leitch A. E. Lucas C. D. Sheldrake T. A. Dorward D. A. (2011). Induction of eosinophil apoptosis by the cyclin-dependent kinase inhibitor AT7519 promotes the resolution of eosinophil-dominant allergic inflammation. PLoS One 6 (9), e25683. 10.1371/journal.pone.0025683 21984938 PMC3184151

[B3] Andreev D. Liu M. Kachler K. Llerins Perez M. Kirchner P. Kolle J. (2021). Regulatory eosinophils induce the resolution of experimental arthritis and appear in remission state of human rheumatoid arthritis. Ann. Rheum. Dis. 80 (4), 451–468. 10.1136/annrheumdis-2020-218902 33148700

[B4] Aravamudan B. Kiel A. Freeman M. Delmotte P. Thompson M. Vassallo R. (2014). Cigarette smoke-induced mitochondrial fragmentation and dysfunction in human airway smooth muscle. Am. J. Physiol. Lung Cell Mol. Physiol. 306 (9), L840–L854. 10.1152/ajplung.00155.2013 24610934 PMC4116419

[B5] Awad A. Yassine H. Barrier M. Vorng H. Marquillies P. Tsicopoulos A. (2014). Natural killer cells induce eosinophil activation and apoptosis. PLoS One 9 (4), e94492. 10.1371/journal.pone.0094492 24727794 PMC3984162

[B6] Bao Y. Ledderose C. Seier T. Graf A. F. Brix B. Chong E. (2014). Mitochondria regulate neutrophil activation by generating ATP for autocrine purinergic signaling. J. Biol. Chem. 289 (39), 26794–26803. 10.1074/jbc.M114.572495 25104353 PMC4175322

[B7] Barnes P. J. (2017). Cellular and molecular mechanisms of asthma and COPD. Clin. Sci. (Lond). 131 (13), 1541–1558. 10.1042/CS20160487 28659395

[B8] Barthel S. R. Johansson M. W. McNamee D. M. Mosher D. F. (2008). Roles of integrin activation in eosinophil function and the eosinophilic inflammation of asthma. J. Leukoc. Biol. 83 (1), 1–12. 10.1189/jlb.0607344 17906117 PMC2859217

[B9] Bonjour K. Palazzi C. Silva T. P. Malta K. K. Neves V. H. Oliveira-Barros E. G. (2022). Mitochondrial population in mouse eosinophils: ultrastructural dynamics in cell differentiation and inflammatory diseases. Front. Cell Dev. Biol. 10, 836755. 10.3389/fcell.2022.836755 35386204 PMC8979069

[B10] Broughton S. E. Dhagat U. Hercus T. R. Nero T. L. Grimbaldeston M. A. Bonder C. S. (2012). The GM-CSF/IL-3/IL-5 cytokine receptor family: from ligand recognition to initiation of signaling. Immunol. Rev. 250 (1), 277–302. 10.1111/j.1600-065X.2012.01164.x 23046136

[B11] Bruno S. R. Kumar A. Mark Z. F. Chandrasekaran R. Nakada E. Chamberlain N. (2021). DRP1-Mediated mitochondrial fission regulates lung epithelial response to allergen. Int. J. Mol. Sci. 22 (20), 11125. 10.3390/ijms222011125 34681784 PMC8540036

[B12] Choi Y. Kim Y. M. Lee H. R. Mun J. Sim S. Lee D. H. (2020). Eosinophil extracellular traps activate type 2 innate lymphoid cells through stimulating airway epithelium in severe asthma. Allergy 75 (1), 95–103. 10.1111/all.13997 31330043

[B13] Choi Y. Le Pham D. Lee D. H. Lee S. H. Kim S. H. Park H. S. (2018). Biological function of eosinophil extracellular traps in patients with severe eosinophilic asthma. Exp. Mol. Med. 50 (8), 104–108. 10.1038/s12276-018-0136-8 30115903 PMC6095846

[B14] Chung K. F. (2013). New treatments for severe treatment-resistant asthma: targeting the right patient. Lancet Respir. Med. 1 (8), 639–652. 10.1016/S2213-2600(13)70128-0 24461667

[B15] Chung K. F. Dixey P. Abubakar-Waziri H. Bhavsar P. Patel P. H. Guo S. (2022). Characteristics, phenotypes, mechanisms and management of severe asthma. Chin. Med. J. Engl. 135 (10), 1141–1155. 10.1097/CM9.0000000000001990 35633594 PMC9337252

[B16] Dente F. L. Bacci E. Bartoli M. L. Cianchetti S. Costa F. Di Franco A. (2010). Effects of oral prednisone on sputum eosinophils and cytokines in patients with severe refractory asthma. Ann. Allergy Asthma Immunol. 104 (6), 464–470. 10.1016/j.anai.2010.04.003 20568377

[B17] De Volder J. Vereecke L. Joos G. Maes T. (2020). Targeting neutrophils in asthma: a therapeutic opportunity? Biochem. Pharmacol. 182, 114292. 10.1016/j.bcp.2020.114292 33080186

[B18] Dewson G. Cohen G. M. Wardlaw A. J. (2001). Interleukin-5 inhibits translocation of Bax to the mitochondria, cytochrome c release, and activation of caspases in human eosinophils. Blood 98 (7), 2239–2247. 10.1182/blood.v98.7.2239 11568012

[B19] Dibbert B. Daigle I. Braun D. Schranz C. Weber M. Blaser K. (1998). Role for Bcl-xL in delayed eosinophil apoptosis mediated by granulocyte-macrophage colony-stimulating factor and interleukin-5. Blood 92 (3), 778–783. 10.1182/blood.v92.3.778.415k38_778_783 9680344

[B20] Didichenko S. A. Spiegl N. Brunner T. Dahinden C. A. (2008). IL-3 induces a Pim1-dependent antiapoptotic pathway in primary human basophils. Blood 112 (10), 3949–3958. 10.1182/blood-2008-04-149419 18768389

[B21] Dimasuay K. G. Berg B. Schaunaman N. Chu H. W. (2023). Role of myeloid cell-specific TLR9 in mitochondrial DNA-induced lung inflammation in mice. Int. J. Mol. Sci. 24 (2), 939. 10.3390/ijms24020939 36674451 PMC9864555

[B22] Druilhe A. Letuve S. Pretolani M. (2003). Glucocorticoid-induced apoptosis in human eosinophils: mechanisms of action. Apoptosis 8 (5), 481–495. 10.1023/a:1025590308147 12975579

[B23] Dworski R. Simon H. U. Hoskins A. Yousefi S. (2011). Eosinophil and neutrophil extracellular DNA traps in human allergic asthmatic airways. J. Allergy Clin. Immunol. 127 (5), 1260–1266. 10.1016/j.jaci.2010.12.1103 21315435 PMC3085562

[B24] Ederlé C. Charles A.-L. Khayath N. Poirot A. Meyer A. Clere-Jehl R. (2019). Mitochondrial function in Peripheral Blood Mononuclear Cells (PBMC) is enhanced, together with increased reactive oxygen species, in severe asthmatic patients in exacerbation. J. Clin. Med. 8 (10), 1613. 10.3390/jcm8101613 31623409 PMC6833034

[B25] Eisner V. Picard M. Hajnoczky G. (2018). Mitochondrial dynamics in adaptive and maladaptive cellular stress responses. Nat. Cell Biol. 20 (7), 755–765. 10.1038/s41556-018-0133-0 29950571 PMC6716149

[B26] Elmore S. (2007). Apoptosis: a review of programmed cell death. Toxicol. Pathol. 35 (4), 495–516. 10.1080/01926230701320337 17562483 PMC2117903

[B27] Esteves P. Blanc L. Celle A. Dupin I. Maurat E. Amoedo N. (2021). Crucial role of fatty acid oxidation in asthmatic bronchial smooth muscle remodelling. Eur. Respir. J. 58 (5), 2004252. 10.1183/13993003.04252-2020 33833033

[B28] Garcia C. de Oliveira M. C. Verlengia R. Curi R. Pithon-Curi T. C. (2003). Effect of dexamethasone on neutrophil metabolism. Cell Biochem. Funct. 21 (2), 105–111. 10.1002/cbf.1002 12736898

[B29] Gardai S. J. Hoontrakoon R. Goddard C. D. Day B. J. Chang L. Y. Henson P. M. (2003). Oxidant-mediated mitochondrial injury in eosinophil apoptosis: enhancement by glucocorticoids and inhibition by granulocyte-macrophage colony-stimulating factor. J. Immunol. 170 (1), 556–566. 10.4049/jimmunol.170.1.556 12496443

[B30] Geijsen N. Koenderman L. Coffer P. J. (2001). Specificity in cytokine signal transduction: lessons learned from the IL-3/IL-5/GM-CSF receptor family. Cytokine Growth Factor Rev. 12 (1), 19–25. 10.1016/s1359-6101(00)00019-8 11312115

[B31] Germic N. Fettrelet T. Stojkov D. Hosseini A. Horn M. P. Karaulov A. (2021). The release kinetics of eosinophil peroxidase and mitochondrial DNA is different in association with eosinophil extracellular trap formation. Cells 10 (2), 306. 10.3390/cells10020306 33546138 PMC7913244

[B32] Håkansson L. Heinrich C. Rak S. Venge P. (1997). Priming of eosinophil adhesion in patients with birch pollen allergy during pollen season: effect of immunotherapy. J. Allergy Clin. Immunol. 99 (4), 551–562. 10.1016/s0091-6749(97)70084-8 9111502

[B33] Hogan S. P. Rosenberg H. F. Moqbel R. Phipps S. Foster P. S. Lacy P. (2008). Eosinophils: biological properties and role in health and disease. Clin. Exp. Allergy 38 (5), 709–750. 10.1111/j.1365-2222.2008.02958.x 18384431

[B34] Ilmarinen P. Moilanen E. Kankaanranta H. (2014). Mitochondria in the center of human eosinophil apoptosis and survival. Int. J. Mol. Sci. 15 (3), 3952–3969. 10.3390/ijms15033952 24603536 PMC3975377

[B35] Ilmarinen-Salo P. Moilanen E. Kinnula V. L. Kankaanranta H. (2012). Nitric oxide-induced eosinophil apoptosis is dependent on mitochondrial permeability transition (mPT), JNK and oxidative stress: apoptosis is preceded but not mediated by early mPT-dependent JNK activation. Respir. Res. 13 (1), 73. 10.1186/1465-9921-13-73 22920281 PMC3495716

[B36] Johansson M. Mosher D. (2013). Integrin activation states and eosinophil recruitment in asthma. Front. Pharmacol. 4 (33), 33. 10.3389/fphar.2013.00033 23554594 PMC3612688

[B37] Jones N. Vincent E. E. Felix L. C. Cronin J. G. Scott L. M. Hole P. S. (2020). Interleukin-5 drives glycolysis and reactive oxygen species-dependent citric acid cycling by eosinophils. Allergy 75 (6), 1361–1370. 10.1111/all.14158 31856334

[B38] Kankaanranta H. Lindsay M. A. Giembycz M. A. Zhang X. Moilanen E. Barnes P. J. (2000). Delayed eosinophil apoptosis in asthma. J. Allergy Clin. Immunol. 106 (1 Pt 1), 77–83. 10.1067/mai.2000.107038 10887309

[B39] Kano G. Almanan M. Bochner B. S. Zimmermann N. (2013). Mechanism of Siglec-8-mediated cell death in IL-5-activated eosinophils: role for reactive oxygen species-enhanced MEK/ERK activation. J. Allergy Clin. Immunol. 132 (2), 437–445. 10.1016/j.jaci.2013.03.024 23684072 PMC4042061

[B40] Kikly K. K. Bochner B. S. Freeman S. D. Tan K. B. Gallagher K. T. D'Alessio K. J. (2000). Identification of SAF-2, a novel siglec expressed on eosinophils, mast cells, and basophils. J. Allergy Clin. Immunol. 105 (6 Pt 1), 1093–1100. 10.1067/mai.2000.107127 10856141

[B41] Koranteng J. Raby K. Dixey P. Lai S. L. Howarth P. Chung K. F. (2023). Role of eosinophil mitochondrial function in severe eosinophilic asthma. Eur. Respir. Soc. Int. Congr. 10.1183/13993003.congress-2023.PA552

[B42] Kuo C. S. Pavlidis S. Loza M. Baribaud F. Rowe A. Pandis I. (2017). T-helper cell type 2 (Th2) and non-Th2 molecular phenotypes of asthma using sputum transcriptomics in U-BIOPRED. Eur. Respir. J. 49 (2), 1602135. 10.1183/13993003.02135-2016 28179442

[B43] Lazarus S. C. Chinchilli V. M. Rollings N. J. Boushey H. A. Cherniack R. Craig T. J. (2007). Smoking affects response to inhaled corticosteroids or leukotriene receptor antagonists in asthma. Am. J. Respir. Crit. Care Med. 175 (8), 783–790. 10.1164/rccm.200511-1746OC 17204725 PMC1899291

[B44] LeMessurier K. S. Rooney R. Ghoneim H. E. Liu B. Li K. Smallwood H. S. (2020). Influenza A virus directly modulates mouse eosinophil responses. J. Leukoc. Biol. 108 (1), 151–168. 10.1002/JLB.4MA0320-343R 32386457 PMC7859173

[B45] Liu Q. Wu J. Zhang X. Li X. Wu X. Zhao Y. (2021). Circulating mitochondrial DNA-triggered autophagy dysfunction via STING underlies sepsis-related acute lung injury. Cell Death Dis. 12 (7), 673. 10.1038/s41419-021-03961-9 34218252 PMC8254453

[B46] Lommatzsch M. Klein M. Stoll P. Virchow J. C. (2019). Impact of an increase in the inhaled corticosteroid dose on blood eosinophils in asthma. Thorax 74 (4), 417–418. 10.1136/thoraxjnl-2018-212233 30315084

[B47] Lood C. Blanco L. P. Purmalek M. M. Carmona-Rivera C. De Ravin S. S. Smith C. K. (2016). Neutrophil extracellular traps enriched in oxidized mitochondrial DNA are interferogenic and contribute to lupus-like disease. Nat. Med. 22 (2), 146–153. 10.1038/nm.4027 26779811 PMC4742415

[B48] Lopez A. F. Sanderson C. J. Gamble J. R. Campbell H. D. Young I. G. Vadas M. A. (1988). Recombinant human interleukin 5 is a selective activator of human eosinophil function. J. Exp. Med. 167 (1), 219–224. 10.1084/jem.167.1.219 2826636 PMC2188822

[B49] Luo H. R. Loison F. (2008). Constitutive neutrophil apoptosis: mechanisms and regulation. Am. J. Hematol. 83 (4), 288–295. 10.1002/ajh.21078 17924549

[B50] Mabalirajan U. Dinda A. K. Kumar S. Roshan R. Gupta P. Sharma S. K. (2008). Mitochondrial structural changes and dysfunction are associated with experimental allergic asthma. J. Immunol. 181 (5), 3540–3548. 10.4049/jimmunol.181.5.3540 18714027

[B51] Maret M. Ruffie C. Letuve S. Phelep A. Thibaudeau O. Marchal J. (2009). A role for Bid in eosinophil apoptosis and in allergic airway reaction. J. Immunol. 182 (9), 5740–5747. 10.4049/jimmunol.0800864 19380821

[B52] Mesnil C. Raulier S. Paulissen G. Xiao X. Birrell M. A. Pirottin D. (2016). Lung-resident eosinophils represent a distinct regulatory eosinophil subset. J. Clin. Invest. 126 (9), 3279–3295. 10.1172/JCI85664 27548519 PMC5004964

[B53] Meyer L. Leymarie O. Chevalier C. Esnault E. Moroldo M. Da Costa B. (2017). Transcriptomic profiling of a chicken lung epithelial cell line (CLEC213) reveals a mitochondrial respiratory chain activity boost during influenza virus infection. PLoS One 12 (4), e0176355. 10.1371/journal.pone.0176355 28441462 PMC5404788

[B54] Michaeloudes C. Abubakar-Waziri H. Lakhdar R. Raby K. Dixey P. Adcock I. M. (2021). Molecular mechanisms of oxidative stress in asthma. Mol. Asp. Med. 85, 101026. 10.1016/j.mam.2021.101026 34625291

[B55] Michaeloudes C. Bhavsar P. K. Mumby S. Chung K. F. Adcock I. M. (2017). Dealing with stress: defective metabolic adaptation in chronic obstructive pulmonary disease pathogenesis. Ann. Am. Thorac. Soc. 14 (Suppl. ment_5), S374–S82. 10.1513/AnnalsATS.201702-153AW 29161091 PMC5711272

[B56] Michaeloudes C. Bhavsar P. K. Mumby S. Xu B. Hui C. K. M. Chung K. F. (2020). Role of metabolic reprogramming in pulmonary innate immunity and its impact on lung diseases. J. Innate Immun. 12 (1), 31–46. 10.1159/000504344 31786568 PMC6959099

[B57] Milara J. Morell A. Roger I. Montero P. Cortijo J. (2023). Mechanisms underlying corticosteroid resistance in patients with asthma: a review of current knowledge. Expert Rev. Respir. Med. 17 (8), 701–715. 10.1080/17476348.2023.2255124 37658478

[B58] Morshed M. Yousefi S. Stockle C. Simon H. U. Simon D. (2012). Thymic stromal lymphopoietin stimulates the formation of eosinophil extracellular traps. Allergy 67 (9), 1127–1137. 10.1111/j.1398-9995.2012.02868.x 22764833

[B59] Mukherjee M. Lacy P. Ueki S. (2018). Eosinophil extracellular traps and inflammatory pathologies-untangling the web. Front. Immunol. 9, 2763. 10.3389/fimmu.2018.02763 30534130 PMC6275237

[B60] Nutku E. Aizawa H. Hudson S. A. Bochner B. S. (2003). Ligation of Siglec-8: a selective mechanism for induction of human eosinophil apoptosis. Blood 101 (12), 5014–5020. 10.1182/blood-2002-10-3058 12609831

[B61] Ora J. Calzetta L. Matera M. G. Cazzola M. Rogliani P. (2020). Advances with glucocorticoids in the treatment of asthma: state of the art. Expert Opin. Pharmacother. 21 (18), 2305–2316. 10.1080/14656566.2020.1807514 32808828

[B62] Park Y. M. Bochner B. S. (2010). Eosinophil survival and apoptosis in health and disease. Allergy Asthma Immunol. Res. 2 (2), 87–101. 10.4168/aair.2010.2.2.87 20358022 PMC2846745

[B63] Pazdrak K. Moon Y. Straub C. Stafford S. Kurosky A. (2016). Eosinophil resistance to glucocorticoid-induced apoptosis is mediated by the transcription factor NFIL3. Apoptosis 21 (4), 421–431. 10.1007/s10495-016-1226-5 26880402 PMC4769953

[B64] Peachman K. K. Lyles D. S. Bass D. A. (2001). Mitochondria in eosinophils: functional role in apoptosis but not respiration. Proc. Natl. Acad. Sci. U. S. A. 98 (4), 1717–1722. 10.1073/pnas.98.4.1717 11172017 PMC29323

[B65] Porter L. Toepfner N. Bashant K. R. Guck J. Ashcroft M. Farahi N. (2018). Metabolic profiling of human eosinophils. Front. Immunol. 9, 1404. 10.3389/fimmu.2018.01404 30013547 PMC6036296

[B66] Prakash Y. Pabelick C. M. Sieck G. C. (2017). Mitochondrial dysfunction in airway disease. Chest 152 (3), 618–626. 10.1016/j.chest.2017.03.020 28336486 PMC5812762

[B67] Prazma C. M. Bel E. H. Price R. G. Bradford E. S. Albers F. C. Yancey S. W. (2019). Oral corticosteroid dose changes and impact on peripheral blood eosinophil counts in patients with severe eosinophilic asthma: a *post hoc* analysis. Respir. Res. 20 (1), 83. 10.1186/s12931-019-1056-4 31053134 PMC6499981

[B68] Previte D. M. O'Connor E. C. Novak E. A. Martins C. P. Mollen K. P. Piganelli J. D. (2017). Reactive oxygen species are required for driving efficient and sustained aerobic glycolysis during CD4+ T cell activation. PLoS One 12 (4), e0175549. 10.1371/journal.pone.0175549 28426686 PMC5398529

[B69] Raby K. L. Michaeloudes C. Tonkin J. Chung K. F. Bhavsar P. K. (2023). Mechanisms of airway epithelial injury and abnormal repair in asthma and COPD. Front. Immunol. 14, 1201658. 10.3389/fimmu.2023.1201658 37520564 PMC10374037

[B70] Reddy P. H. (2011). Mitochondrial dysfunction and oxidative stress in asthma: implications for mitochondria-targeted antioxidant therapeutics. Pharmaceuticals 4 (3), 429–456. 10.3390/ph4030429 21461182 PMC3066010

[B71] Robin E. D. Wong R. (1988). Mitochondrial DNA molecules and virtual number of mitochondria per cell in mammalian cells. J. Cell Physiol. 136 (3), 507–513. 10.1002/jcp.1041360316 3170646

[B72] Rosenberg H. F. Dyer K. D. Foster P. S. (2013a). Eosinophils: changing perspectives in health and disease. Nat. Rev. Immunol. 13 (1), 9–22. 10.1038/nri3341 23154224 PMC4357492

[B73] Rosenberg H. F. Dyer K. D. Foster P. S. (2013b). Eosinophils: changing perspectives in health and disease. Nat. Rev. Immunol. 13 (1), 9–22. 10.1038/nri3341 23154224 PMC4357492

[B74] Rothenberg M. E. Hogan S. P. (2006). THE EOSINOPHIL. Annu. Rev. Immunol. 24 (1), 147–174. 10.1146/annurev.immunol.24.021605.090720 16551246

[B75] Samarasinghe A. E. Melo R. C. Duan S. LeMessurier K. S. Liedmann S. Surman S. L. (2017). Eosinophils promote antiviral immunity in mice infected with influenza A virus. J. Immunol. 198 (8), 3214–3226. 10.4049/jimmunol.1600787 28283567 PMC5384374

[B76] Segal M. Niazi S. Simons M. P. Galati S. A. Zangrilli J. G. (2007). Bid activation during induction of extrinsic and intrinsic apoptosis in eosinophils. Immunol. Cell Biol. 85 (7), 518–524. 10.1038/sj.icb.7100075 17549073

[B77] Shen Z. J. Esnault S. Schinzel A. Borner C. Malter J. S. (2009). The peptidyl-prolyl isomerase Pin1 facilitates cytokine-induced survival of eosinophils by suppressing Bax activation. Nat. Immunol. 10 (3), 257–265. 10.1038/ni.1697 19182807 PMC2847832

[B78] Silveira J. S. Antunes G. L. Kaiber D. B. da Costa M. S. Marques E. P. Ferreira F. S. (2019). Reactive oxygen species are involved in eosinophil extracellular traps release and in airway inflammation in asthma. J. Cell Physiol. 234 (12), 23633–23646. 10.1002/jcp.28931 31180592

[B79] Sivertson K. L. Seeds M. C. Long D. L. Peachman K. K. Bass D. A. (2007). The differential effect of dexamethasone on granulocyte apoptosis involves stabilization of Mcl-1L in neutrophils but not in eosinophils. Cell Immunol. 246 (1), 34–45. 10.1016/j.cellimm.2007.05.003 17573055 PMC2213750

[B80] Song D. J. Cho J. Y. Lee S. Y. Miller M. Rosenthal P. Soroosh P. (2009). Anti-Siglec-F antibody reduces allergen-induced eosinophilic inflammation and airway remodeling. J. Immunol. 183 (8), 5333–5341. 10.4049/jimmunol.0801421 19783675 PMC2788790

[B81] Travers J. Rothenberg M. E. (2015). Eosinophils in mucosal immune responses. Mucosal Immunol. 8 (3), 464–475. 10.1038/mi.2015.2 25807184 PMC4476057

[B82] Trian T. Benard G. Begueret H. Rossignol R. Girodet P.-O. Ghosh D. (2007a). Bronchial smooth muscle remodeling involves calcium-dependent enhanced mitochondrial biogenesis in asthma. J. Exp. Med. 204 (13), 3173–3181. 10.1084/jem.20070956 18056286 PMC2150973

[B83] Trian T. Benard G. Begueret H. Rossignol R. Girodet P. O. Ghosh D. (2007b). Bronchial smooth muscle remodeling involves calcium-dependent enhanced mitochondrial biogenesis in asthma. J. Exp. Med. 204 (13), 3173–3181. 10.1084/jem.20070956 18056286 PMC2150973

[B84] Ueki S. Melo R. C. Ghiran I. Spencer L. A. Dvorak A. M. Weller P. F. (2013). Eosinophil extracellular DNA trap cell death mediates lytic release of free secretion-competent eosinophil granules in humans. Blood 121 (11), 2074–2083. 10.1182/blood-2012-05-432088 23303825 PMC3596967

[B85] Ventura-Clapier R. Garnier A. Veksler V. (2008). Transcriptional control of mitochondrial biogenesis: the central role of PGC-1alpha. Cardiovasc Res. 79 (2), 208–217. 10.1093/cvr/cvn098 18430751

[B86] Walsh G. M. (2013). Eosinophil apoptosis and clearance in asthma. J. Cell Death 6, 17–25. 10.4137/JCD.S10818 25278777 PMC4147767

[B87] Wenzel S. Ford L. Pearlman D. Spector S. Sher L. Skobieranda F. (2013). Dupilumab in persistent asthma with elevated eosinophil levels. N. Engl. J. Med. 368 (26), 2455–2466. 10.1056/NEJMoa1304048 23688323

[B88] Wenzel S. Wilbraham D. Fuller R. Getz E. B. Longphre M. (2007). Effect of an interleukin-4 variant on late phase asthmatic response to allergen challenge in asthmatic patients: results of two phase 2a studies. Lancet 370 (9596), 1422–1431. 10.1016/S0140-6736(07)61600-6 17950857

[B89] Whetstone C. E. Ranjbar M. Omer H. Cusack R. P. Gauvreau G. M. (2022). The role of airway epithelial cell alarmins in asthma. Cells 11 (7), 1105. 10.3390/cells11071105 35406669 PMC8997824

[B90] Wiese A. V. Duhn J. Korkmaz R. U. Quell K. M. Osman I. Ender F. (2023). C5aR1 activation in mice controls inflammatory eosinophil recruitment and functions in allergic asthma. Allergy 78 (7), 1893–1908. 10.1111/all.15670 36757006

[B91] Wu G. Zhu Q. Zeng J. Gu X. Miao Y. Xu W. (2019). Extracellular mitochondrial DNA promote NLRP3 inflammasome activation and induce acute lung injury through TLR9 and NF-κB. J. Thorac. Dis. 11 (11), 4816–4828. 10.21037/jtd.2019.10.26 31903272 PMC6940233

[B92] Xu W. Ghosh S. Comhair S. A. Asosingh K. Janocha A. J. Mavrakis D. A. (2016a). Increased mitochondrial arginine metabolism supports bioenergetics in asthma. J. Clin. investigation 126 (7), 2465–2481. 10.1172/JCI82925 PMC4922712 27214549

[B93] Xu W. Ghosh S. Comhair S. A. Asosingh K. Janocha A. J. Mavrakis D. A. (2016b). Increased mitochondrial arginine metabolism supports bioenergetics in asthma. J. Clin. Invest. 126 (7), 2465–2481. 10.1172/JCI82925 27214549 PMC4922712

[B94] Youle R. J. van der Bliek A. M. (2012). Mitochondrial fission, fusion, and stress. Science 337 (6098), 1062–1065. 10.1126/science.1219855 22936770 PMC4762028

[B95] Yousefi S. Gold J. A. Andina N. Lee J. J. Kelly A. M. Kozlowski E. (2008). Catapult-like release of mitochondrial DNA by eosinophils contributes to antibacterial defense. Nat. Med. 14 (9), 949–953. 10.1038/nm.1855 18690244

[B96] Yousefi S. Simon D. Simon H. U. (2012). Eosinophil extracellular DNA traps: molecular mechanisms and potential roles in disease. Curr. Opin. Immunol. 24 (6), 736–739. 10.1016/j.coi.2012.08.010 22981682

[B97] Zhang Y. Do D. C. Hu X. Wang J. Zhao Y. Mishra S. (2020). CaMKII oxidation regulates cockroach allergen-induced mitophagy in asthma. J. Allergy Clin. Immunol. 147, 1464–1477.e11. 10.1016/j.jaci.2020.08.033 32920093 PMC8544000

